# Recognition of Gallotannins and the Physiological Activities: From Chemical View

**DOI:** 10.3389/fnut.2022.888892

**Published:** 2022-06-01

**Authors:** Hua-Feng He

**Affiliations:** ^1^College of Pharmacy, Jining Medical University, Jining, China; ^2^Tea Research Institute, Chinese Academy of Agricultural Sciences, Hangzhou, China

**Keywords:** gallotannins, structural characteristic, resource distribution, physiological activities, industrial application

## Abstract

Gallotannins, characterized with the glycosidic core and galloyl unit, are seemed as vital components of hydrolyzable tannins. Benefit from the more and more discoveries of their bioactivities and edibility, application of gallotannins in food industry, pharmacy industry, and other fields is increasing. Inheriting previous study achievements, chemical structure of gallotannins was illustrated and degradation as well as synthetic routes to gallotannins were summarized. On this basis, distribution in the nature also including the distinction of gallotannins was discussed. More than that, activities involving in antioxidant, anti-inflammatory, enzyme inhibitions, protein binding, and so on, as well as applications in the field of food industry, biopharmaceutical science, agricultural production, etc., were combed. Finally, improvement of bioavailability, chemical modification of the structure, and accurate determination of new gallotannins were pointed out to be the orientation in the future.

## Introduction

It had been well known that diet rich in fruits, vegetables, and nuts is beneficial to health. To trace these phenomena to their sources, phytochemicals contained in these products play an essential role in the health benefits. Phytochemicals, especially plant-derived polyphenolic compounds, have been proved to possess a wide range of pharmacological properties. Among the multitudinous food ingredients, tannins occupy an important place. Originally, “tannin” was coined by Seguin to describe the substances present in vegetable extracts (1). In the nature, tannins have been found in a variety of plants, fruits, wines, forages, and tea. With a large number of hydroxyl or other functional groups located on the skeleton, tannins are capable of forming cross-linkages with proteins and other macromolecules. Usually, tannins could covalently bind with alkaloids, gelatin, and proteins to form coagulation. Due to the precipitation with protein, tannins were introduced to leather tanning initially. Roughly divided into hydrolyzable tannins (HTs) and condensed tannins, tannins were reported with multiple health benefits, e.g., antioxidants, anti-inflammatory, anticancer (2–4). Belonging to HTs, gallotannins (GTs) and ellagitannins (ETs) are the most widely occurring tannins. Thereinto, GTs are one of the most often underlined components. In general, GTs are seemed as the simplest HTs (5). Comparatively, GTs were more likely to interact with lipid membranes for its more hydrophobicity (6). Characterized with the glycosidic core and galloyl unit, GTs are present in nearly all the plants in the nature. Herbals, which were rich in gallo- or ellagitannins, had been used to improve vascular health and many other diseases since antiquity. For the nutraceutical and prophylactic potentials, GTs have been of scientific interest. Lots of fruitful pioneer study had been documented (Table 1). According to the results of clinical, interventional, and animal *in vivo* studies, it could be clearly concluded that GT-containing products possessed anti-inflammatory potential (7). It seems that GTs are beneficial to reduce risk of chronic diseases connected with the elevated inflammatory state, particularly cardiovascular diseases (CVDs), (8) type 2 diabetes, (9) inflammatory bowel diseases (IBD), (10) and other gastrointestinal tract pathological conditions.

**TABLE 1:** Reviews related to physiological activities of gallotannins (GTs) documented in recent 5 years.

Biological activity	Conclusion	References
Anti-inflammatory	Directly application of GTs containing preparations on inflamed tissues exhibit impact on inflammatory processes. However, structural changes mediated by gut microbiota during the transit through gastrointestinal tract should be referred. Coupled with dietary fiber, GTs may result in intestinal health benefits.	(7, 11)
Anti-bacteria	GTs inhibit growth of intestinal flora by preventing bacterial adhesion to the intestinal epithelium and prevent coliform diarrhea by inhibiting the bacterial enterotoxins and channels involved in the secretion of electrolytes and water into the lumen.	(12)
Anti-tumor	Represented by GTs, phytochemicals contained in *Mangifera indica*, *Punica granatum*, possess strong anti-tumor activity, inhibit breast cancer cell growth, proliferation, migration and invasion as well as trigger apoptosis and cell cycle arrest.	(13, 14)
Modulation of hemostasis system	Tannins, including GTs, modify the activity of platelets, coagulation, the fibrinolysis system, and endothelium, reduce the risk of thromboembolism.	(15)
Interference of metabolites	Due to the capacity to trap ammonia and H_2_S, and to modify the composition and populations of the microbiota, tannins reduce the production of potentially toxic metabolites in farm animals, which would be benefit to the quality of animal products, i.g., organoleptic qualities of meat and milk.	(16)
Pharmacology and toxicology	Main pharmacological activities as well as toxicity of tannins were discussed.	(4)

Despite study for centuries, there are still many confused conclusion and unresolved contradictions waiting to be figured out. For instance, structural definition and scientific classification are still ambiguous. Also, functional activities of GTs were multifarious. Thus, it is necessary to comb the superficial cognition about GTs.

### Chemical Characterization of Gallotannins

Before discussing the biosynthetic pathway to GTs, it appears indispensable to insert a few comments on the structural principles and conventional definitions used in this field. First of all, it should be legible with regard to the chemical structure of GTs. Structurally surrounded by several galloyl units, GTs are with a polyol as the core, most often glucose. On the other hand, GTs are identified as polygalloyl esters of glucose. Apart from glucose, diverse polyol, catechin, or triterpenoid cores could be bounded to the galloyl unit (17). Such GTs, containing glucitol core, were identified from Red maple (*Acer rubrum*) (18). Unnatural GTs, taking 2-C-(hydroxymethyl)-branched aldoses as the central polyol, were designed and synthesized (19). Employing 1,5-anhydroalditol and inositol as the cyclic polyol cores, Machida et al. designed and obtained 14 unnatural gallotannin derivatives (20).

**FIGURE 1:**
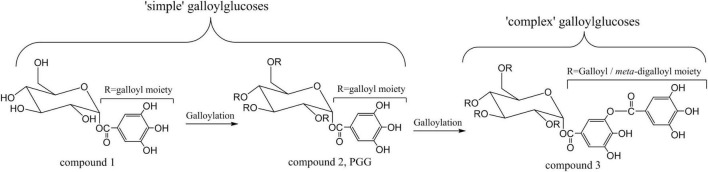
The structural illustration of gallotannins.

According to Niemeta and Gross (21), GTs could further be subdivided into “simple” and “complex” galloylglucoses. As a natural product, 1-O-galloyl-β-*D*-glucopyranose (compound 1, Figure 1), the simplest member of GTs, had been isolated and characterized more than a century ago. According to the number of galloyl groups, there are MoGG, one galloyl group; DiGG, two galloyl groups; TriGG, three galloyl groups; and TeGG, four galloyl groups. Fully substitution of galloyl to glucose would lead to 1,2,3,4,6-penta-O-galloyl-β-*D*-glucopyranose (PGG) (compound 2, Figure 1) (22), which seemed as the prototypical “complex” galloylglucoses (23). Sequentially, gallyol units can be grafted through depside bonds to form “complex” galloylglucoses with high molecular weight (MW). 2-*O*-digalloyl-1,3,4,6-tetra-*O*-galloyl-β-*D*-glucose (compound 3, hexagalloylglucose), the typical representative of these “complex” galloylglucoses, is given in Figure 1. One point to be emphasized was that gallic acid now combined with phenolic hydroxyls *via* a depsidic bond. As known, chemical properties are significantly different between the aliphatic OH-groups of the glucose and phenolic hydroxyl groups. It had been proved that the depside bonds between galloyl units are considerably easier to broken than the core ester linkages (6). In wake of the presence of an esteric linkage between two galloy moieties, GTs are also considered to be depsidic metabolites.

### Degradation and Synthesis of Gallotannins

The degradation of GTs consists of a series of enzymatic catalyzed reactions. These enzymes refer to tannase, decarboxylase, pyrogallol 1,2-dioxygenase, pyrogallol-phloroglucinol isomerase, phloroglucinol reductase, dihydrophloroglucinol hydrolase, phenol oxidase, etc. First, cleavage of depside bonds takes place. Following, hydrolysis of the ester bond was undergone to yield gallic acid. Then, catalyzed by gallic acid decarboxylase, decarboxylation would be taken place on gallic acid to form pyrogallol. Ulteriorly, pyrogallol would convert to open loop secondary metabolites, e.g., pyruvic acid, and finally enters the Krebs cycle (24).

Also, GTs could be easily degraded by bacteria, fungi, and yeasts (25, 26). As reported, microorganisms, present in the rumen and the distal portion of the monogastric intestine of the ruminants, were involved in the hydrolysis of ester and depside linkages of GTs. During the procedure, glucose and gallic acid were released. Furthermore, gallic acid would continue degrade to pyrogallol and phloroglucinol and, ultimately, to acetate and butyrate. In the light of the present situation, biodegradation of GTs is still in an incipient stage. For the treatment with large-scale applications, especially in food, fodder, medicine, and tannery effluent, there is still a long way to go.

When it turns to the synthesis of GTs, biosynthesis would be first discussed. Derived from an intermediate of 5-dehydroshikimate, formation of gallic acid was reported to be through a shikimate pathway (27). Catalyzed by enzyme extracts from oak leaves, esterification between gallic acid and glucose was conducted to form β-glucogallin (1-O-galloyl-β-D-glucose) (28). During this procedure, uridine diphosphate (UDP) glucose served as an activated substrate. In association with the biosynthetic pathway, β-glucogallin is presumed to be the first intermediate and a key metabolite in the synthesis of GTs. Enzyme studies conducted by Grundhofer et al. indicated that β-glucogallin was required as a principal acyl donor for the biosynthesis of GTs (29). Without any other cofactors, the transformation of β-glucogallin, formed *in situ*, to di- and trigalloylglucose, was catalyzed by these enzymes. β-glucogallin was deemed to function not only as acyl acceptor, but also as efficient acyl donor (24). Demonstrated by immunohistochemical studies, leaf mesophyll cell walls were the typical site of origin and deposition of hydrolyzable tannins.

With extreme regularity, substitution of glucose hydroxyls was not randomly distributed in these conversions. Thus, the metabolic sequence followed the following order: β-glucogallin → 1,6-digalloylglucose → 1,2,6-trigalloylglucose → 1,2,3,6-tetragalloylglucose → 1,2,3,4,6-pentagalloylglucose. Accompanied with the formation of PGG, generally considered to be a simple galloyl glucose ester, the first stage for the biosynthetic pathway of GTs is completed. In all the plants, the pathway to PGG had been regarded to be identical. The transition from “simple” galloylglucoses to “complex” GTs is marked by the attachment of further galloyl residues to PGG to obtain the *meta*-depside groups (30). Taking PGG as the core, further galloylation reactions were conducted at the phenolic hydroxyl groups. During the second step, high-molecular metabolites containing up to 10 galloyl residues were formed (31).

On the other hand, for the reason of natural materials in short supply and difficulties in the isolation and purification, study work and the practical utilization of GTs were limited. Because of this, chemical synthesis of GTs was documented unremittingly. The first total chemical syntheses of two GTs, hexagalloylglucose and decagalloylglucose, was reported by Sylla et al. (32). Employing commercially 1,2:5,6-di-O-isopropylidene-α-*D*-glucofuranose as the starting material, the first galloyl moiety was introduced at the O3 position of the glucose unit. Under mild non-acidic Steglich-type conditions, successive galloylation reaction was taken place to give the corresponding 3-*O*-galloylated *D*-glucofuranose. Mediated by palladium in tetrahydrofuran (THF) at room temperature for 24 h, the hexagalloyl-β-glucopyranoses, PGG, would be obtained after the final hydrogenolysis of the fully protected hexagalloyl-*D*-glucopyranoses (32). Employing benzyl-protected gallic acid chloride as the substrate, reaction conditions that lead to high anomeric selectivity for the synthesis of GT were investigated. Accordingly, there is a significant correlation between solvent for the reaction and α:β selectivity. After screening, the highest selectivity was found when acetonitrile (CH_3_CN) was used as a solvent. Meanwhile, dichloromethane (CH_2_Cl_2_) was demonstrated to accelerate the rate of mutarotation relative to the rate of condensation to the ester (33). Starting from slight modification of methyl gallate, coupling with galloylation sequentially, alkyl GTs, which are structurally related with natural compounds, were synthesized (34). And then, protective effects were investigated.

### Distribution and Distinction

Various galloyl-glucopyranoses occur naturally in lots of plants. Representatively, occurrence of GTs was found in the extracts of Chinese galls (*Rhus semialata*) and Turkish galls (*Gallae turcicea*). Also, presence of GTs in plants, such as *Paeonia* (23) and *Schinus terebinthifolius*, the leaves of Sicillian sumac (*Rhus coriora* L.) and the common smoke tree (*Cotinus coggygria Scop.*) were at sufficient levels to allow direct isolation and utilization (17). Among 42 edible beans, red sword bean (*Canavalia gladiata*) was found to have the highest content of GTs (35). Also, first report of the presence of GTs in nutshell of camellia tree (*Camellia oleifera C*. Abel) was disclosed by He et al. (36). From the byproducts of mango, barks, kernels, leaves, etc., GTs were identified and quantitated (37).

As to the refined structure, the various sources resulted in GTs with different chemical structures. As well, the concentration of GTs varies with plant genotype, tissue developmental stage, and environmental conditions (38). Several inconsistent theories had been developed regarding the chemical composition of GTs distributed in different plants. Although the contents varied greatly, the presence of GTs was fairly consistent across a range of cultivars. Under the circumstances, general assays were much preferred to elucidate specific structures of GTs. Chelation with metal ions and the formation of complexes guaranteed the detection of GTs by UV–Vis spectroscopy. Advances in describing GTs structural variation using high-performance liquid chromatography–mass spectrometry (HPLC–MS) had been made (39). Undoubtedly, simple colorimetric assays for the measurement of tannins and other phenolics facilitated many ecological studies of GTs, whereas the appropriate standard was crucial for the assay. Quantitatively, huge errors might be obtained if an inappropriate standard was used, for example, the commonly used quebracho tannin. Stumbling blocks for controlled studies on specific GTs were still existed due to the lack of commercially available standards. It is delightful, with the extraction of crude tannin and additional purification, a growing number of standards had been prepared by more and more researchers in a related field. Taking the degree of hydrolysis into consideration, amounts of free gallic acids were also indeterminate in quantity. According to the report, ratio of glucose to gallic acid was 1–9 or 10 in oriental sumac GTs in contrast to 1–5 or 6 in Turkish gall nuts (39).

Certainly, extraction would make a big difference in analysis of GTs. Despite the lack of commercially available standards, pioneer articles related to extraction, optimization, identification, and quantification of GTs were documented constantly. In respect to separation of GTs from mango kernels, extract process comprised of degreasing of seeds, extraction with aqueous acetone [80% (v/v)], and use of high-speed countercurrent chromatography for purification (40). With the crude extract in hand, subsequent purification was applied by being partitioned against ethyl acetate. According to the degree of galloylation (tetra-O-galloylglucose to deca-O-galloylglucose), solvent system employing hexane/ethyl acetate/methanol/water [0.5:5:1:5 (v/v/v/v)] was used in the head-to-tail mode to elute tannins. It had been proved that the using of hexane for the extraction of mango kernel fat would not affect GTs in respect to the profile and contents (41). In contrast, when methanol was used, significant changes were observed, which might result from the methanolysis of GTs to PGG. Methyl gallate and PGG would be yielded due to simultaneous extraction and methanolytic conversion of GTs. Degradation of higher GTs took place when methanol was used merely for the extraction of mango seeds. All these constituted important facets with respect to the utilization of GTs present in byproducts derived from mango fruit processing.

The rapid development of instruments as well as the innovation in analytical methods facilitates the discovery of new novel GTs. Meanwhile, improved detection accuracy guaranteed the detection of GTs in a wider range of plant species. Based on the flow-injection analysis–electrospray ionization–ion trap–tandem mass spectrometry (FIA-ESI-IT-MS-MS) and matrix-assisted laser desorption/ionization-time-of-flight mass spectrometry (MALDI-TOF-MS), a rapid and reliable analytical approach was proposed to investigate the full file of HTs present in the extracts of *Astronium* species and evidenced the existence of GTs in *Astronium* genus first (42). According to Boulekbache-Makhlouf, 18 GTs were present in fruit of *Eucalyptus globulus* growing in Algeria (43). By means of high-performance liquid chromatography–electrospray ionization mass spectrometry (HPLC–ESI-MS) method, assisted by diode array detection, 14 GTs could be isolated from birch (*Betula pubescens*) leaves (44). With the occurrence of absorption maxima between 275 and 280 nm on UV spectra, LC-MS molecular ion and fragment ion peaks, combining with NMR survey to the proton and carbon signals, five GTs were identified from *Sapria himalayana f. albovinosa* in Myanmar (45). Study result revealed that monogalloyl to hexagalloyl hexosides comprised the GTs film. Characterized by MALDI-TOF-MS, relying on the alteration of cationization reagents, Cs^+^, K^+^, and Na^+^, a different spectrum of Chinese GTs was given (46).

### Physiological Activities of Gallotannins

There is one consensus to be emphasized that GT structural details are fateful for many of their biological effects. Equipped with the amount of hydroxyl groups (-OH) as well as the high molecular complexity, GTs were documented with several pro- and antiphysiological effects, such as antioxidant (47), antidiabetic (9), precipitating proteins (48), and antimicrobial (49). Theoretically, cooperative effects of hydrogen bonding and hydrophobic association were responsible for the GT–protein and GT–phospholipid interactions (48). Likewise, hydrogen bonding was pointed to be the predominant effect in the interactions between GTs and sugars.

#### Antioxidant

It is important to note that health benefits mentioned above all associated with the antioxidant capacity of GTs. Studies showed that these properties were result of the number of the galloyl groups and their position (50). Consistent with the number of the galloyl groups, interactions with proteins and ability to scavenge 2,2-diphenyl-1-picrylhydrazyl (DPPH) radicals improved (24). According to the study, antioxidative activity of GTs increased proportionally to the number of galloyl moieties (51). Determined antioxidative capacities of GTs on lipid peroxidation were PGG > TeGG > TGG > DiGG > GA (45). With regard to the placement of the galloyl groups, a study to investigate the inhibitory effects of 1,2, 6-,1,3, 6-, and 3,4,6-TriGG on lipid peroxidation in mitochondria and microsomes of the liver was conducted (52). The comparative antioxidative effect came to the conclusion: 1,3,6-TriGG > 1,2,6-TriGG > 3,4,6-TriGG. In contrast to inactive of 1,3,4,6-TeGG on the affinity of GTs to galloyltransferase, strong effect was shown by 1,2,3,6-TeGG (53). On the other hand, the galloyl groups of GTs were detected as the hydrophobic sites, through which interaction with aliphatic side chains of amino acids was established (29). Diversely, evaluation of antioxidant, antimicrobial, and antibiofilm activities indicated that type of sugar played an important role on the inhibitory effect (19).

#### Anti-inflammatory

Anti-inflammatory effects were shown by GT-containing products, especially in treatment with inflammatory bowel disease (IBD) and cardiovascular diseases (CVDs). In ulcerative colitis (UC), a primary component of IBD, GTs also exhibited significant advantages (54). Protective effect on dextran sulfate sodium (DSS)-induced colitis was witnessed by treatment with corilagin, a gallotannin present in many medical plants. Acting on the nuclear factor-kappa B (NF-κB) pathway, corilagin could mitigate colon inflammatory responses and the apoptosis of intestinal epithelial cells. By a self-assembly technique, Turkish galls GTs (TGTs)-Fe^III^ microcapsules were prepared (55). *Ex vivo* and *in vivo* adhesion experiments showed that accumulation of the microcapsules was more likely to occur on the inflammatory surface and UC symptoms were effectively alleviated. To get a better sense of the postulated effectiveness, new perspective was given (7). Expression and activity of inducible nitric oxide synthase (iNOS), as well as the production of nitric oxide (NO), were inhibited by PGG (24). As a result, the production of proinflammatory cytokines and the adhesive molecules in endothelial cells would be decreased. Also, it would reduce the aggregation of thrombocytes and induce relaxation of contracted aortic rings. As documented, GTs could dose-dependently decrease gene expression and production of iNOS. As the result, lipopolysaccharide (LPS)-induced NO production was significantly reduced in macrophages. In other words, GTs isolated from Euphorbia species (*Euphorbiaceae*) showed an inhibitory effect on the LPS-induced inflammatory reaction (56).

#### Sensory Reinforcement

Being the predominant substance contributed to the orosensory sensation of food, GTs were employed to investigate the aversive behavior on astringency of foods by naïve mice (57). Undergoing a one-bottle preference test, ingestion of serial dilutions of GTs by inbred mice was assessed. The result indicated concentration-dependently inhibition of GTs on daily drink consumption. For this reason, drink intake was far predominant at night (circadian rhythm). Sometimes, astringency was tasted when we drink tea. A combination of tannin and oral salivary protein was responsible for the sensual experience.

#### Antidiabetic

The aggregation propensity of GTs equipped them with the potential to be used as antidiabetic drugs. With the numerous hydroxyl groups located on GTs skeleton, gallotannin-enzyme interactions were witnessed at the pancreatic lipase (PL)-colipase complex interface (58). According to molecular docking, GTs were provided with strong affinity toward the enzyme-substrate complex (uncompetitive inhibition). IC50 22.4 and 64.6 μM, respectively, implied GTs to be effective inhibitors, which endowed GTs to be used to treat dyslipidemias and obesity. Moreover, potential beneficial activity toward diabetes and metabolic syndrome was exhibited by PGG (24). Applying to adipocytes, stimulation of glucose transport and suppression of adipocyte differentiation were caused by PGG. Administration of glucitol-core containing gallotannins (GCGs)-enriched red maple leaves extract would be a benefit to colon-derived short-chain fatty acids (SCFAs) production and metabolic improvement (18). In turn, this supported the utilization of red maple GTs as a dietary ingredient for preventing obesity and related metabolic diseases. As a poly (ADP-ribose) glycohydrolase (PARG) inhibitor, protection of glomerular damage was suggested with the treatment of GTs. Through aggregation, GTs originated from Aleppo oak exerted the nature as non-specific promiscuous α-amylase inhibitors (9). Being inhibitor of α-glucosidase, GTs were reported to possess antidiabetic properties (51). As a hallmark for apoptotic cell death, protection in poly (ADP-ribose) polymerase (PARP) cleavage signified the protective role of GTs in cell death signaling (59). The result portended GTs to be used as an alternative approach to ameliorate the development of streptozotocin-induced diabetic nephropathy.

#### Induction of Apoptosis

Gallotannins were proved to be potent inhibitors and disruptors of a series of bacteria (19). As documented, the outer membrane conferred bacterial resistance to microbicide (60). Benefiting from the strong affinity for iron, GTs showed inhibitory activities against the growth of bacteria. In response to the shin-whitening effect, GTs isolated from Chinese galls showed inhibitory activity against tyrosinase and inhibited the biosynthesis of melanin, which is related to hyperpigmentation (61). In a study on the antitumor mechanism, cytotoxicity against HepG2 and Chang hepatocellular carcinoma cells was exhibited by GTs (21). By inhibiting cell adhesion and suppressing cell repair motility, GTs would induce apoptosis of hepatocellular carcinoma cells.

### Application of Gallotannins

#### Food Processing

Hypothetically, GTs would be oxidized to reactive quinone by laccase (Lac) in laccase-mediator system (LMS) (62). Compared to the direct catalyzation of Lac, quinone could attack acid-swollen collagen (ASC) more easily. This would result in an efficient crosslinking and related performance of ASC. For the reinforcement, GTs were introduced as a mediator for application in ASC films. In the food industry, especially meat processing, LMS containing GTs would be option to modify collagenous material greenly. High selectivity on the regulation of the growth of bacteria was showed by penta-, hexa-, and heptagalloylglucose (63). In contrast to the inhibition of several pathogenic bacteria, growth of non-pathogenic lactic acid bacteria is not affected. This predicted the application of GTs as biopreservatives in foods. Fabricating insoluble gallotannin-pectin complexes, GTs could be bound by pectin (64). According to report, maximum mass ratios of combination GT to pectin for hydrochloric acid-extracted pectin (HEP) and chelating agent-extracted pectin (ChEP) were 0.28 and 0.47, respectively. It leads to ChEP was a better additive in beverage containing GTs, while HEP had advantage in emulsifying stability. In a dose-dependent manner, GTs improved collagen synthesis and reduced matrix metalloproteinase-1 (MMP-1) expression. In UVB-irradiated human cells, GTs downregulated MMP-1 levels through the extracellular signal-regulated kinase/c-Jun N-terminal kinase (ERK/JNK) signaling pathway (65). Also, glutathione rather than transforming growth factor β-1, which would induce fibrosis, would be increased by GTs.

#### Food Package

To extend shelf-life and reduce the risk from foodborne bacteria, many efforts had been tried on packing films/coasting. For the sake of the food packaging legislation of many regions and given the inactivation or evaporation of the antimicrobial agents under the harsh conditions (high temperature, pressure, and shear forces), allyl isothiocyanate or nanosilver films were circumscribed for use in food packing. Furthermore, the negative impact on organoleptic qualities of food was also an important reason. Thus, packaging film that was universally approved and commercialized available for antimicrobial of food was desired. For the proven antibacterial properties and food contact permit, GTs were introduced into food packaging films (66). The thermal process would trigger the release of free gallic acid from GTs and the high concentration of gallic acid would lead to an increase of antioxidant ability (67). Proudly, this character guaranteed GTs the inhibitory effect on lipid oxidation and convinced the use in the food package.

#### Plant Protection

Furthermore, as protein-binding agents, GTs are still often referred in the field of plant–insect interactions. Benefiting from the inherent affinity for proteins, GTs endowed the defense against pathogenic microbes to plants chemically. In addition, in blocking the infection of viral pathogens against insects and herbivores, GTs also played an important role. Both of these advantages might be *via* protein binding and enzyme inactivation caused by GTs. For instance, GTs in oak could reduce the infectivity of naturally occurring nuclear polyhedrosis viruses in gypsy moth caterpillars (68–70). Against *Ralstonia solanacearum* (*R. solanacearum*), potent antibacterial activity, *in vitro* and *in vivo*, was shown by GTs extracted from *Sedum takesimense*. Even more, GTs displayed broad-spectrum activity against various plant-pathogenic bacteria. This made it possible for the use of GTs as natural bactericides for the control of tomato bacterial wilt (71). Antibacterial activities *in vitro* showed that the strongest was against *R. solanacearum*. As documented, 1,2,6-tri-O-galloyl-β-D-glucopyranose isolated from *Terminalia chebula* fruit exhibited antibacterial action against multidrug-resistant uropathogenic *Escherichia coli* (*E. coli*) (72). Possibly, efflux pump inhibitory activity may be one of the mechanisms. Besides, revealed by Tuominen and Salminen that contents of polyphenol, GTs, e.g., differed significantly among ontogenic phases. According to this, evaluation of the role of polyphenols in plant–herbivore interactions would be feasible and render it plausible for the planned best collection times of *Geranium sylvaticum* with compound isolation purpose (73).

#### Phylaxiology

To some extent, GTs could be identified as the molecular basis of red wine and green tea for their benefits in prevention of cardiovascular disease and antisecretory action (74). According to documents, the calcium-activated Cl^–1^ channel (CaCC) plays an important role in cell physiology, in particular the treatment of hypertension, diarrhea, and cystic fibrosis. Being a modulator, GTs strongly inhibited TMEM16A with IC_50_ was 10.0 μM, which indicated potential utility for the treatment mentioned above.

### Safety and Toxicity

Despite the diverse physiological activities as well as the extensive application, especially in food industry, double-edged sword of GTs should not be neglected. Intake of GTs might lead to damage of intestine and digestion, especially in susceptible species, e.g., sheep (75). In severe case, failure of kidney and liver would be resulted. Experimental data revealed that synthetic GTs, methyl 2,3,4,6-tetra-O-galloyl-α-D-glucoside (G4Glc), methyl 2,3,4,6-tetra-O-galloyl-α-Dmannoside (G4Man), and methyl 2,3,4-tri-O-galloyl-α-L-rhamnoside (G3Rham), possessed significant antioxidant activities (24). Whereas, very low genotoxic effect on human peripheral blood mononuclear cells (PBMCs) was manifested. Moreover, profiting from the protection of these compounds, damage induced by hydrogen peroxide or Fe^2+^ on DNA would be alleviated considerably. Based on these results, it could be concluded that synthetic GTs represent non-toxic agents, which implied the potential biomedical applications of GTs. At another level, it would be useful for the subsequent design of new antioxidants. Because of the binding with protein of GTs, bioavailability of both the proteins and polyphenols would be reduced. Previous studies suggested that vegetable tannins possess a high binding affinity to proline-rich proteins (76). Hydrophobic stacking of the planar phenolic ring against the pyrrolidine ring of the proline was pointed out to be the primary between PGG and proline-rich peptides. The molecular weight as well as structural flexibility of GTs was related to the ability of precipitation of protein. Depending on GTs concentration and nutrient levels, it would be beneficial for mammals for the ability to bind with proteins in the guts (41).

## Conclusion and Prospect

Stand on the foundation of pioneer study, perception of GTs was refreshed. The precise chemical structure about GTs was illustrated to define the structural characteristics accurately. The degradation of GTs, with the presence of enzymes, was summarized cumulatively. The result would be beneficial for the understanding of the synthesis of GTs. Subsequently, distribution as well as the distinction of GTs distributed in different resources was proposed, which would be constructive for the utilization. Sequentially, the advantages of GTs, performed on antioxidant, anti-inflammatory, sensory reinforcement, antidiabetic, induction of apoptosis, etc., were combed systematically. All these endowed the good prospects of GTs for scale application. Following, applications of GTs in the food process, food package, plant protection, and phylaxiology were enumerated. In view of the dual property of phytochemicals, safety and toxicity of GTs were also referred.

Despite amount of excellent study and sufficient attention had been paid, accompanied with massive literature reports, insight into GTs is still at the very beginning stage. Concerted efforts are still needed to achieve more extensive and in-depth utilization of GTs.

### Improvement of the Bioavailability

Due to the limited bioavailability of GTs, intake of GT-containing products orally can influence immune response at the level of the gastrointestinal tract. Modulating effects on the gut microbiota composition would be affected as well. More accurate delivery to affected areas as well as more efficient absorption and utilization is still in front of the urgent need to solve the problem.

### Chemical Modification of the Structure

At the level of action mechanism, structure–function relationship indicated among polyphenolated templates, naturally occurring GTs may be not the optimal protein recognition agents in fact (77). For this reason, modification of the structure of GTs with the help of chemical synthesis was desired. From another perspective, assays for detection of GTs require the access to standard monomeric substance and chemical synthesis would be a powerful means. Development of the chemical synthetic methodology to construct GTs awaits to be optimized.

### Accurate Determination of New Gallotannins

With the advantage of analytical instruments, it becomes possible for the determination of GTs with more accuracy and faster. Taking ultramodern instruments and technique into application, characterization of monomers and analogs of GTs remains to be clarified systemically. Also, content of trace in new resources would be no longer the obstacle for verification. As a result, it is worth looking forward to see the increase of the membership of GTs family.

## Author Contributions

The author confirms being the sole contributor of this work and has approved it for publication.

## Conflict of Interest

The author declares that the research was conducted in the absence of any commercial or financial relationships that could be construed as a potential conflict of interest.

## Publisher’s Note

All claims expressed in this article are solely those of the authors and do not necessarily represent those of their affiliated organizations, or those of the publisher, the editors and the reviewers. Any product that may be evaluated in this article, or claim that may be made by its manufacturer, is not guaranteed or endorsed by the publisher.
